# Effectiveness of Baduanjin exercise for improving the mental health of university students: a systematic review and meta-analysis of randomized controlled trials

**DOI:** 10.3389/fpsyg.2026.1825503

**Published:** 2026-05-15

**Authors:** Jiangmei Chen, Qingfeng Geng, Siheng Xie, Qiang Zhang, Liqiang Yu

**Affiliations:** 1 School of Humanities & Management, Fujian University of Traditional Chinese Medicine, Fuzhou, Fujian, China; 2 School of New Huadu Business, Minjiang University, Fuzhou, Fujian, China; 3 School of Economics and Management, Anqing Normal University, Anqing, Anhui, China; 4 Department of Nursing, Fuzhou University Affiliated Provincial Hospital, Fuzhou, Fujian, China; 5 Department of Surgical Intensive Care Unit, Fuzhou University Affiliated Provincial Hospital, Fuzhou, Fujian, China

**Keywords:** Baduanjin, mental health, meta-analysis, sleep quality, students, systematic review

## Abstract

**Purpose:**

This study aimed to systematically evaluate the effects of Baduanjin exercise on mental health, sleep quality and fatigue among university students, explore the influence of intervention parameters on the outcomes, and assess the methodological quality of relevant randomized controlled trials (RCTs). In doing so, it addresses the current evidence gap regarding this non-pharmacological intervention, which remains fragmented despite the limitations of conventional mental health approaches.

**Methods:**

Systematic searches were conducted in both English and Chinese databases to identify RCTs comparing Baduanjin intervention with routine care measures, covering all literature published up to January 15, 2026. Statistical analyses were performed using Review Manager 5.4 and Stata/SE 15.1, and subgroup analyses were conducted to explore how variations in intervention parameters influenced the measured outcomes.

**Results:**

A total of 36 RCTs involving 3,233 university students were included in the systematic review, of which 35 studies with 2,846 participants were eligible for meta-analysis. Baduanjin practice significantly improved overall mental health (Symptom Checklist-90, SCL-90: 95% CI: −0.27 to −0.10, *p* < 0.0001), alleviated depressive symptoms (Self-Rating Depression Scale, SDS: 95% CI: −5.67 to −3.00, *p* < 0.0001), anxiety (Self-Rating Anxiety Scale, SAS: 95% CI: −6.30 to −1.73, *p* = 0.0006), reduced negative mood states (Profile of Mood States, POMS: 95% CI: −10.73 to −3.57, *p* < 0.0001), perceived stress (Perceived Stress Scale/Chinese Perceived Stress Scale, PSS/CPSS: 95% CI: −0.77 to −0.08, *p* = 0.02), and fatigue (Fatigue Scale-14, FS-14: 95% CI: −1.55 to −0.46, *p* < 0.0001), while enhancing sleep quality (Pittsburgh Sleep Quality Index, PSQI: 95% CI: −3.41 to −1.74, p < 0.0001). Subgroup analyses suggested practicing Baduanjin three sessions per week may be most beneficial for general mental health (SCL-90), and a duration of 12 weeks or less may be more effective for reducing depressive symptoms (SDS) based on current evidence. Follow-up assessment indicated that improvements in sleep and stress were not sustained after intervention cessation. The overall methodological quality of included studies was moderate.

**Conclusion:**

Regular Baduanjin practice effectively improves mental health, sleep quality, and subjective fatigue among university students. A frequency of a three-times-weekly schedule and a duration of up to 12 weeks appears to be associated with more favorable effects on selected outcomes. Given its low cost, ease of standardization, and scalability, Baduanjin may be considered as a potentially valuable complementary intervention for college mental health promotion. Future research should enhance methodological rigor to validate these findings and investigate the long-term maintenance mechanisms of therapeutic effects.

## Introduction

Within the university context, students commonly encounter stressors such as academic pressure, economic burdens, and lifestyle transitions, which substantially elevate their risk of developing mental health issues and sleep disorders (Zhao and Zhang, 2024). A global umbrella review involving over 8.7 million university students revealed that 41.09% of participants suffered from sleep disorders, 35.41% reported mild depressive symptoms, and 40.21% exhibited mild anxiety symptoms (Paiva et al., 2025). These problems are not merely transient physiological or psychological reactions; they may progress to chronic mental illnesses like generalized anxiety disorder and even increase the risk of chronic somatic conditions, including metabolic syndrome and cardiovascular disease (Marta et al., 2025). Consequently, exploring preventive and intervention measures for university students’ mental health and safeguarding their mental and sleep wellbeing have become pressing priorities.

Pharmacological treatment of mental disorders (e.g., antidepressants and anxiolytics) is often associated with adverse effects, such as sleep disturbance, sexual dysfunction, and weight gain (Zhang et al., 2024), while cognitive behavioral therapy (CBT) is limited by inadequate accessibility, high costs, and prolonged waiting times for service (Zhu et al., 2024). In contrast to pharmacological interventions, exercise therapy offers the advantages of high safety and fewer complications, making it a crucial approach for improving psychological health (Huang K. et al., 2024; Liu L. Y. et al., 2025). As a time-honored traditional Chinese health exercise, Baduanjin exhibits unique and significant advantages in enhancing mental health due to its characteristics of slow and coherent movements, profound cultural connotation, and the integration of body and mind (Dong et al., 2023). Its low intensity, high-inclusivity, and holistic regulatory properties distinguish it significantly from high-intensity physical exercise, rendering it more suitable for meeting the comprehensive health needs of university students (Liang et al., 2024). Compared with yoga, which involves complex postures and poses challenges for beginners (Martínez-Calderon et al., 2023), or mindfulness meditation, which requires high levels of sustained concentration and is difficult to adapt to fragmented schedules (Lothes et al., 2025), Baduanjin features simple, tranquil movements with a low learning threshold, which are more accessible and practical. Therefore, it is better suited to the realistic characteristics of university students, such as tight schedules and substantial differences in physical fitness levels (García-Pérez et al., 2025).

Currently, several systematic reviews have suggested that Baduanjin exerts a significant positive effect on improving mental health and sleep quality. A comprehensive study indicated that Baduanjin can effectively reduce depressive and anxiety symptoms, alleviate subjective fatigue, and enhance sleep quality in patients with chronic diseases, individuals with cognitive impairment, and adult experiencing psychological distress (Liu J. H. et al., 2023; Liu H. Y. et al., 2023). Another meta-analysis including 11 RCTs further confirmed that compared with conventional interventions, Baduanjin could significantly relieve negative emotions in patients with type 2 diabetes who suffer from psychological distress (Luo et al., 2023). In recent years, there has been a growing number of individual RCTs focusing on university students, and the results generally demonstrate that Baduanjin can alleviate anxiety and depression and improve overall mental health. However, existing studies still have obvious limitations. On the one hand, most studies focus solely on a single psychological dimension (e.g., depression) or sleep quality in isolation, lacking an integrated assessment of multiple related psychological indicators such as anxiety, stress, and fatigue. On the other hand, there is no high-quality systematic meta-analysis to explore the dose–response relationship between intervention parameters of Baduanjin (including total intervention duration, weekly frequency, and single session duration) and effect size. Identifying the optimal intervention plan is a core prerequisite for developing evidence-based exercise prescriptions.

To address the shortcomings of existing research, this study focuses on the university students’ mental health problems, systematically explores the mechanism and practical effect of Baduanjin exercise, and conducts a high-quality systematic review and meta-analysis. Firstly, it comprehensively evaluated the overall effects of Baduanjin on university students’ mental health, sleep quality, and fatigue relief. Secondly, it analyzes the impact of key intervention parameters on effectiveness to identify the optimal intervention combination. Finally, it assesses the methodological quality of the included studies using the revised Cochrane Risk of Bias Tool (ROB 2.0). Based on the latest high-quality empirical evidence, this study aims to provide theoretical support for universities to scientifically integrate Baduanjin into their mental health promotion systems and offer a reference for formulating evidence-based and individualized intervention strategies in related fields.

## Methods

This systematic review and meta-analysis was conducted in strict adherence to the Preferred Reporting Items for Systematic Reviews and Meta-analysis (PRISMA) 2020 statement (Page et al., 2021). The complete PRISMA checklist is provided in the Supplementary Table 1.

### Search strategy

A comprehensive systematic search was performed across six English databases, including the Cochrane Library, PubMed, Embase, Web of Science, PsycINFO, and Ovid MEDLINE, as well as four Chinese databases (SinoMed, CNKI, VIP Information, and Wanfang Data). The search period spanned from the inception of each database to January 15, 2026. The search strategy was conducted based on three core concepts of “Baduanjin,” “university students,” and “mental health.” To ensure the comprehensive retrieval of all relevant RCTs, a combination of subject headings and free-text terms was employed for searching across all databases. Detailed search strategies for Web of Science and CNKI are provided in the Appendix.

### Study selection criteria

#### Inclusion criteria

(1) Study design: RCTs; (2) Study population: University students (without limitations regarding gender, age, ethnicity, or physical health status); (3) Interventions: The experimental group received Baduanjin exercise as the primary intervention, while the control group received routine interventions (e.g., usual study/lifestyle, health education); (4) Multi-group studies: Only subgroups that met the aforementioned inclusion criteria were included; (5) Outcomes: At least one validated psychometric assessment instrument for mental health evaluation. Primary outcomes involved general mental health (Symptom Checklist-90, SCL-90), depressive symptoms (Self-Rating Depression Scale, SDS), anxiety symptoms (Self-Rating Anxiety Scale, SAS), sleep quality (Pittsburgh Sleep Quality Index, PSQI), and fatigue status (Fatigue Scale-14, FS-14). Secondary outcomes comprised negative emotional states (Profile of Mood States, POMS), perceived stress (Perceived Stress Scale/Chinese Perceived Stress Scale, PSS/CPSS), Depression Anxiety Stress Scales-21 (DASS-21), as well as relevant subscale dimensions of the above instruments.

#### Exclusion criteria

(1) Duplicate publications or studies with overlapping data; (2) Interventions combined with other qigong practices or similar traditional Chinese exercises; (3) Studies with unbalanced baseline characteristics or missing key baseline data; (4) Studies with unavailable full-text or insufficient data to extract relevant outcomes.

### Study selection and data extraction

Two independent researchers conducted literature screening in two sequential stages: initially, titles and abstracts were screened to exclude obviously ineligible studies, followed by full-text review of the remaining studies to make the final eligibility determination. Any discrepancies arising from the screening process were resolved through face-to-face discussion between the two researchers; if no consensus could be achieved, a third independent researcher was invited to provide the final adjudication. To ensure data accuracy, two independent researchers completed the data extraction work separately. The extracted data covered five core dimensions: (1) Basic study characteristics (title, author, publication year, country); (2) Population characteristics (sample size, age, gender distribution, health status); (3) Intervention details (intervention duration, weekly frequency, session length, delivery mode, control group interventions); (4) Outcome measures; (5) Adverse events and follow-up information. Consistent with the literature screening process, discrepancies encountered during data extraction were resolved through discussion and consultation with a third independent researcher. A standardized data extraction form was pre-designed to collect all relevant information. To avoid duplicate data inclusion, only endpoint outcome data were extracted from studies with multiple follow-up time points, and duplicate publications derived from identical research cohorts were strictly screened and excluded.

### Risk of bias assessment

Two independent reviewers conducted separate evaluations of the methodological quality for all included RCTs, using the ROB 2.0 (Sterne et al., 2019), which assesses five core domains: (1) Randomization process; (2) Deviations from intended interventions; (3) Missing outcome data; (4) Outcome measurement; (5) Selection of the reported results. In accordance with the predefined judgment criteria of ROB 2.0, each domain was graded as “Low risk,” “Some concerns,” or “High risk” of bias. Following independent assessment, the two researchers cross-validated their findings; any discrepancies were resolved via discussion or, if necessary, arbitration by a third researcher. Microsoft Excel was employed to generate risk-of-bias graphs and summary figures for the visual presentation of methodological quality across all included studies.

### Statistical analyses

Statistical analyses were performed using Review Manager 5.4 and Stata/SE 15.1 software. For continuous outcomes, the mean difference (MD) with 95% confidence interval (CI) served as the effect size. The standardized mean difference (SMD) was adopted for scales with inconsistent versions (PSS/CPSS), as well as FS-14 with inconsistent standardized reference sources across studies. To ensure comparability, all SCL-90 scores reported as total or mean values were uniformly converted to mean scores before meta-analysis. A fixed-effects model was used if heterogeneity was low (*p* ≥ 0.10 and I^2^ ≤ 50%); otherwise, a random-effects model was adopted. The I^2^ statistic was prioritized over the *p*-value when assessing heterogeneity, with clinical heterogeneity also taken into consideration. *p* < 0.05 was statistically significant. Sensitivity analysis was conducted via the sequential exclusion method to verify the robustness of the pooled effect sizes for the primary outcomes. Publication bias was assessed using funnel plots combined with Egger’s linear regression; a *p*-value > 0.05 for Egger’s test suggested no significant publication bias.

## Results

### Literature search and study selection

**Figure 1:**
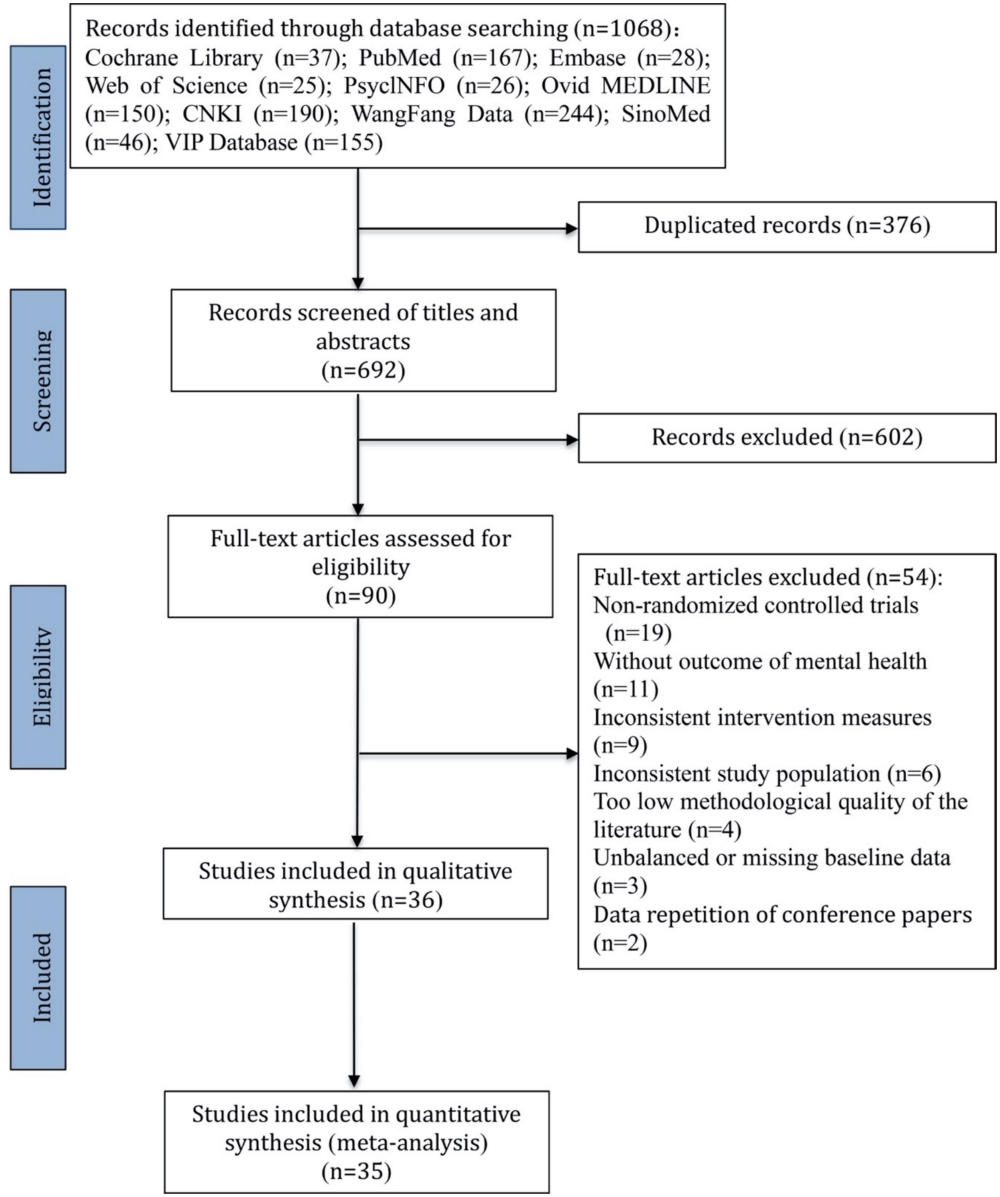
PRISMA flow diagram for study selection.

The literature search and screening procedures are detailed in Figure 1. The initial search yielded 1,068 potentially relevant articles, and 36 studies (Deng, n.d.; Huang, 2025; Li, 2025; Liu N. N. et al., 2025; Zhong, 2025; Alkhatib et al., 2024; Wang Z. P. et al., 2024; Yao et al., 2024; Zhao et al., 2024; Wang, 2025; Wei et al., 2024; Liu et al., 2024; Ding and Tan, 2024; Zhang and Jiang, 2023; Zou et al., 2023; Qin et al., 2023; Zhang, 2023; Meng, 2023; Li et al., 2022; Xiao et al., 2021; Guo, 2021; Wang et al., 2021; Zhou et al., 2020; Li et al., 2020; Tan and Tan, 2020; Ke et al., 2019; Kang et al., 2018; Yan and Wei, 2017; Li, 2017; Li et al., 2015; Zhao and Li, 2014; Xue et al., 2014; Li et al., 2014; Geng, 2013; Mo, 2013; Liu, 2007) were finally included after sequential layer-by-layer screening. Among these, thirty-five studies involving a total of 2,846 subjects met the inclusion criteria for the meta-analysis.

### Characteristics of included studies

#### Participant baseline characteristics

The baseline characteristics of participants across the 36 RCTs are summarized in Table 1. A total of 3,233 university students (including undergraduates and postgraduates) were enrolled, with sample sizes ranging from 30 to 387 per study and a mean age of 18.93–27.87 years. Most studies (*n* = 21) did not impose gender restrictions, while 6 studies (Liu N. N. et al., 2025; Alkhatib et al., 2024; Zhao et al., 2024; Zhang and Jiang, 2023; Kang et al., 2018; Mo, 2013) focused exclusively on female students. Regarding health status, 18 studies (Zhao et al., 2024; Wang, 2025; Liu et al., 2024; Ding and Tan, 2024; Zhang and Jiang, 2023; Meng, 2023; Li et al., 2022; Xiao et al., 2021; Guo, 2021; Wang et al., 2021; Zhou et al., 2020; Li et al., 2020; Yan and Wei, 2017; Li, 2017; Li et al., 2015; Zhao and Li, 2014; Mo, 2013; Liu, 2007) included healthy participants, whereas the remaining 18 studies targeted students with specific conditions, such as mild or more severe depression (Yao et al., 2024; Wei et al., 2024; Qin et al., 2023; Tan and Tan, 2020; Xue et al., 2014; Li et al., 2014), sleep disturbance (Huang, 2025; Li, 2025; Liu N. N. et al., 2025; Zou et al., 2023; Zhang, 2023), and mental sub-health (Geng, 2013). Incomplete reporting was observed for grade (13 studies), sex distribution (17 studies), and age (5 studies).

**Table 1:** Baseline characteristics of participants in included studies.

No.	Study (author, year)	Location (publication language)	Participants	Grade	Health status	Sex (male/female)	Total sample size (C/E)	Age (years)
1	Deng (n.d.)	China (Chinese)	University students	Undergraduate students	Qi-deficiency constitution	C: (26/2) E: (27/2)	57 (28/29)	C: 19–21 E: 19–21
2	Huang (2025)	China (Chinese)	University students	Undergraduate students	Sleep disturbance	C: (13/16) E: (14/15)	58 (29/29)	C: 22.76 ± 2.03 E: 23.17 ± 2.36
3	Li (2025)	China (Chinese)	University students	Undergraduate students	Sleep disturbance	C: (4/11) E: (6/9)	30 (15/15)	C: 21.73 ± 2.05 E: 22.73 ± 1.75
4	Liu N. N. et al. (2025)	China (Chinese)	Female university students	Undergraduate students	Sleep disturbance	C: (0/28) E: (0/28)	56 (28/28)	C: 21 ± 2 E: 22 ± 3
5	Zhong (2025)	China (Chinese)	University students	Undergraduate students	Cervical sub-health status	C: (7/15) E: (7/13)	42 (20/22)	C: 18.75 ± 0.97 E: 19.08 ± 0.99
6	Alkhatib et al. (2024)	China (English)	Female university students (non-Chinese)	Undergraduate and postgraduate students	Menstrual discomfort	C: (0/31) E: (0/31)	62 (31/31)	All: 27.87 ± 5.58
7	Wang Z. P. et al. (2024)	China (English)	University students	Undergraduate students	Physically disadvantaged	C: (25/22) E: (24/22)	93 (47/46)	C: Male 19.18 ± 0.32 Female 18.96 ± 0.11 E: Male 19.21 ± 0.17 Female 19.76 ± 0.26
8	Yao et al. (2024)	China (English)	University students	Second-year undergraduate students	Mild or higher depression	C: (9/31, 4 dropouts) E: (8/32, 1 dropout)	75 (36/39)	C: 19.33 ± 0.66 E: 19.40 ± 0.59
9	Zhao et al. (2024)	China (English)	Female university students	Undergraduate students	Healthy	C: (0/30) E: (0/30)	60 (30/30)	All: 19 ± 1.30
10	Wang (2025)	China (Chinese)	University students	First-year undergraduate students	Healthy	C: (3/23) E: (6/22)	54 (26/28)	C: 18.96 ± 0.72 E: 18.93 ± 0.54
11	Wei et al. (2024)	China (Chinese)	University students	First-year undergraduate students	Mild or higher depression	NR	200 (100/100)	All: 19–21
12	Liu et al. (2024)	China (Chinese)	University students	First- and second-year undergraduate students	Healthy	NR	60 (30/30)	All: 23 ± 1
13	Ding and Tan (2024)	China (Chinese)	University students	Fourth-year undergraduate students	Healthy	C: (5/60) E: (11/59)	135 (65/70)	C: 20.64 ± 0.71 E: 20.83 ± 0.99
14	Zhang and Jiang (2023)	China (English)	Female university students	First-year undergraduate students	Healthy	C: (0/39) E: (0/34)	73 (39/34)	C: 19.16 ± 1.05 E: 19.23 ± 0.98
15	Zou et al. (2023)	China (Chinese)	University students	Undergraduate students	Sleep disturbance	C: (10/9) E: (10/9)	38 (19/19)	C: 20.53 ± 1.93 E: 20.47 ± 1.47
16	Qin et al. (2023)	China (Chinese)	University students	First-year undergraduate students	Mild to moderate depression	NR	80 (40/40)	C: 17.60 ± 0.84 E: 17.50 ± 0.71
17	Zhang (2023)	China (Chinese)	University students	Undergraduate students	Sleep disturbance	C: (8/12) E: (10/10)	40 (20/20)	C: 20.1 ± 0.69 E: 19.9 ± 0.55
18	Meng (2023)	China (Chinese)	University students	Undergraduate and postgraduate students	Healthy	NR	40 (20/20)	NR
19	Li et al. (2022)	China (English)	University students	Undergraduate and postgraduate students	Healthy	C: (106/89) E: (91/101)	387 (192/195)	C: 23 ± 3 E: 24 ± 4
20	Xiao et al. (2021)	China (English)	University students	First, second, and third-year undergraduate students	Healthy	C: (24/10) E: (24/7)	65 (34/31)	C: 19.71 ± 1.77 E: 19.21 ± 1.02
21	Guo (2021)	China (Chinese)	University students	First-year undergraduate students	Healthy	NR	60 (30/30)	NR
22	Wang et al. (2021)	China (Chinese)	University students	First-year undergraduate students	Healthy	NR	200 (100/100)	All: 19–21
23	Zhou et al. (2020)	China (Chinese)	University students	Undergraduate students	Healthy	C: (16/15) E: (14/17)	62 (31/31)	C: 20.35 ± 0.14 E: 20.52 ± 0.14
24	Li et al. (2020)	China (Chinese)	University students	Undergraduate students	Healthy	C: (9/24) E: (7/26)	66 (33/33)	C: 19.70 ± 1.43 E: 19.85 ± 2.39
25	Tan and Tan (2020)	China (Chinese)	University students	Second- and third-year undergraduate students	Mild or higher depression	NR	70 (35/35)	NR
26	Ke et al. (2019)	China (Chinese)	University students	First- and second-year undergraduate students	Cervical-shoulder syndrome	NR	37 (17/20)	C: 19.50 ± 0.60 E: 19.40 ± 0.50
27	Kang et al. (2018)	China (Chinese)	Female university students	Undergraduate students	Primary dysmenorrhea	C: (0/63) E: (0/63)	126 (63/63)	C: 19 ± 1.50 E: 19 ± 1.30
28	Yan and Wei (2017)	China (Chinese)	University students	Undergraduate students	Healthy	NR	100 (50/50)	NR
29	Li (2017)	China (Chinese)	University students	Undergraduate students	Healthy	NR	60 (30/30)	All: 21–22
30	Li et al. (2015)	China (English)	University students	First- and second-year undergraduate students	Healthy	C: (21/84) E: (15/86)	206 (105/101)	C: 20.92 ± 1.15 E: 20.63 ± 1.03
31	Zhao and Li (2014)	China (Chinese)	University students	First-year undergraduate students	Healthy	C: (55/45) E: (53/47)	200 (100/100)	C: 19.50 ± 1.70 E: 19.80 ± 1.20
32	Xue et al. (2014)	China (Chinese)	University students	First-year undergraduate students	Mild depression	C: (5/10) E: (8/11)	34 (15/19)	C: 17–19 E: 17–19
33	Li et al. (2014)	China (Chinese)	University students	Undergraduate students	Mild to moderate depression	C: (8/12) E: (8/12)	40 (20/20)	All: 20–23
34	Geng (2013)	China (Chinese)	University students	Second-year undergraduate students	Psychological sub-health status	NR	47 (24/23)	All: 20.28 ± 0.86
35	Mo (2013)	China (Chinese)	Female university students	Undergraduate students	Healthy	NR	120 (60/60)	All: 17–25
36	Liu (2007)	China (Chinese)	University students	Undergraduate students	Healthy	NR	100 (50/50)	NR

C: Control group; E: Experimental group; NR: Not reported in the study; Qi-deficiency constitution: Traditional Chinese Medicine diagnosis; Age data are presented as mean ± standard deviation or minimum–maximum range.

#### Experiment and control protocols

The details of the intervention protocols are presented in Table 2. All experimental groups received Baduanjin exercise, with variations in key parameters: session duration ranged from 15 to 120 min (most commonly 40–60 min; *n* = 21), weekly frequency from one to seven sessions (predominantly three–five sessions; *n* = 30), and intervention duration from four to 40 weeks (most frequently 12 weeks; *n* = 15). Interventions were primarily delivered in group settings (*n* = 34), with one study (Li et al., 2022) adopting individual practice and one study (Yao et al., 2024) not specifying the delivery mode. Control group interventions included usual study/lifestyle maintenance (*n* = 29), plus health education (Deng, n.d.; Zou et al., 2023; Li et al., 2022) (*n* = 3), concurrent extracurricular exercise (Zhao et al., 2024; Liu et al., 2024; Kang et al., 2018) (*n* = 3), or health education with optional non-Baduanjin exercise (Alkhatib et al., 2024) (*n* = 1).

**Table 2:** Intervention details of included studies.

No.	Study (author, year)	Control intervention	Experimental intervention	Experimental program	Group/ individual	Duration (weeks)	Follow-up (months) and data available	Outcome measures	Adverse event monitoring	Adverse event reporting
1	Deng (n.d.)	Health education + usual study/lifestyle	Health education + Baduanjin	60 min/session, 4 sessions/week	Group	10	1 (NR)	DASS-21	Reported	NR
2	Huang (2025)	Usual study/lifestyle	Baduanjin	60 min/session, 5 sessions/week	Group	10	None	PSQI	NR	NR
3	Li (2025)	Usual study/lifestyle	Baduanjin	80 min/session, 2 sessions/week	Group	12	None	SCL-90, PSQI	NR	NR
4	Liu N. N. et al. (2025)	Usual study/lifestyle	Baduanjin	45 min/session, 5 sessions/week	Group	10	None	PSQI, FS-14	NR	NR
5	Zhong (2025)	Usual study/lifestyle	Baduanjin	45 min/session, 3 sessions/week	Group	12	None	PSQI	NR	NR
6	Alkhatib et al. (2024)	Health education + optional non-Baduanjin exercise guidance	Baduanjin	30 min/session, 7 sessions/week	Group	12	3 (PSS, PSQI)	PSS, PSQI	NR	No adverse events occurred
7	Wang Z. P. et al. (2024)	Usual study/lifestyle	Baduanjin	60 min/session, 3 sessions/week	Group	16	None	SCL-90	NR	NR
8	Yao et al. (2024)	Usual study/lifestyle	Baduanjin	15 min/session, 7 sessions/week	Not specified	4	None	SAS, SDS, PSQI	NR	NR
9	Zhao et al. (2024)	Extracurricular exercise at the same time as Baduanjin	Baduanjin	60 min/session, 5 sessions/week	Group	16	None	SCL-90	NR	NR
10	Wang (2025)	Usual study/lifestyle	Baduanjin	60–90 min/session, 3 sessions/week	Group	12	None	SCL-90	NR	NR
11	Wei et al. (2024)	Usual study/lifestyle	Baduanjin	90 min/session, 1 session/week	Group	18	None	PSQI	NR	NR
12	Liu et al. (2024)	Extracurricular exercise at the same time as Baduanjin	Baduanjin	45 min/session, 3 sessions/week	Group	16	None	SCL-90	NR	NR
13	Ding and Tan (2024)	Usual study/lifestyle	Baduanjin	40 min/session, 3 sessions/week	Group	40	None	SAS, SDS	NR	NR
14	Zhang and Jiang (2023)	Usual study/lifestyle	Baduanjin	60 min/session, 3 sessions/week	Group	12	None	SCL-90	NR	NR
15	Zou et al. (2023)	Health education + usual study/lifestyle	Health education + Baduanjin	60 min/session, 4 sessions/week	Group	10	None	PSQI	NR	NR
16	Qin et al. (2023)	Usual study/lifestyle	Baduanjin	60 min/session, 3 sessions/week	Group	12	None	PSQI	NR	NR
17	Zhang (2023)	Usual study/lifestyle	Baduanjin	60 min/session, 5 sessions/week	Group	8	None	DASS-21, PSQI	NR	NR
18	Meng (2023)	Usual study/lifestyle	Baduanjin	90 min/session, 3 sessions/week	Group	12	None	SDS, SCL-90	NR	NR
19	Li et al. (2022)	Health education + usual study/lifestyle	Baduanjin	45 min/session, ≥5 sessions/week	Primarily individual practice	12	None	CAS, PWBS	NR	No adverse events occurred
20	Xiao et al. (2021)	Usual study/lifestyle	Baduanjin	90 min/session, 3 sessions/week	Group	12	2 (CPSS, SAS, UCLA-LS, FIS-14)	CPSS, SAS, UCLA-LS, FIS-14	NR	NR
21	Guo (2021)	Usual study/lifestyle	Baduanjin	45 min/session, 3 sessions/week	Group	12	None	SCL-90	NR	NR
22	Wang et al. (2021)	Usual study/lifestyle	Baduanjin	90 min/session, 3 sessions/week	Group	18	None	SCL-90, POMS	NR	NR
23	Zhou et al. (2020)	Usual study/lifestyle	Baduanjin	30 min/session, 5 sessions/week	Group	6	None	SDS, PSQI, FS-14	NR	NR
24	Li et al. (2020)	Usual study/lifestyle	Baduanjin	30 min/session, 7 sessions/week	Group	9	None	FS-14	NR	NR
25	Tan and Tan (2020)	Usual study/lifestyle	Baduanjin	40 min/session, 5 sessions/week	Group	36	None	SDS	NR	NR
26	Ke et al. (2019)	Usual study/lifestyle	Baduanjin	60–90 min/session, 5 sessions/week	Group	10	None	SAS, SDS	NR	NR
27	Kang et al. (2018)	Extracurricular exercise at the same time as Baduanjin	Baduanjin	60 min/session, 3 sessions/week	Group	12	None	SCL-90	NR	NR
28	Yan and Wei (2017)	Usual study/lifestyle	Baduanjin	40 min/session, ≥5 sessions/week	Group	12	None	SCL-90	NR	NR
29	Li (2017)	Usual study/lifestyle	Baduanjin	50 min/session, 5 sessions/week	Group	8	None	PSQI	NR	NR
30	Li et al. (2015)	Usual study/lifestyle	Baduanjin	60 min/session, 5 sessions/week	Group	12	3 (SCL-90, CPSS, POMS, PSQI)	SCL-90, CPSS, POMS, PSQI	NR	No adverse events occurred
31	Zhao and Li (2014)	Usual study/lifestyle	Baduanjin	90 min/session, 5 sessions/week	Group	12	None	POMS	NR	NR
32	Xue et al. (2014)	Usual study/lifestyle	Baduanjin	120 min/session, 1 session/week	Group	8	None	SCL-90	NR	NR
33	Li et al. (2014)	Usual study/lifestyle	Baduanjin	40 min/session, 5 sessions/week	Group	36	None	SDS	NR	NR
34	Geng (2013)	Usual study/lifestyle	Baduanjin	30 min/session, 3 sessions/week	Group	4	None	SCL-90	NR	NR
35	Mo (2013)	Usual study/lifestyle	Baduanjin	60 min/session, 3 sessions/week	Group	24	None	SCL-90, POMS	NR	NR
36	Liu (2007)	Usual study/lifestyle	Baduanjin	90 min/session, 5 sessions/week	Group	12	None	SCL-90, POMS	NR	NR

NR: Not reported in the study; min: minutes; Outcome measures are presented as abbreviations, full names are provided in the footnote below: 1. DASS-21: Depression Anxiety Stress Scales-21; 2. PSQI: Pittsburgh Sleep Quality Index; 3. SCL-90: Symptom Checklist-90; 4. FS-14: Fatigue Scale-14; 5. PSS: Perceived Stress Scale; 6. CPSS: Chinese version of Perceived Stress Scale; 7. SAS: Self-Rating Anxiety Scale; 8. SDS: Self-Rating Depression Scale; 9. POMS: Profile of Mood States; 10. CAS: Coronavirus Anxiety Scale;11. PWBS: Psychological Well-Being Scale; 12. UCLA-LS: UCLA Loneliness Scale; 13. FIS: Feelings of Inadequacy Scale.

#### Follow-up and outcome measures

Follow-up assessments were performed in four studies (Deng, n.d.; Alkhatib et al., 2024; Xiao et al., 2021; Li et al., 2015). Among these, three studies (Alkhatib et al., 2024; Xiao et al., 2021; Li et al., 2015) reported outcome data at two- or three- months post-intervention. A range of validated assessment tools was used to measure outcomes. SCL-90 (*n* = 16) was the most frequently employed, followed by PSQI (*n* = 13), SDS (*n* = 7), POMS (*n* = 5), SAS (*n* = 4), PSS/CPSS (*n* = 3), FS-14 (*n* = 3), DASS-21 (*n* = 2), Coronavirus Anxiety Scale (CAS, *n* = 1), and Psychological Well-Being Scale (PWBS, *n* = 1). Regarding safety outcomes, adverse event monitoring was reported in 1 study (Deng, n.d.), 3 studies (Alkhatib et al., 2024; Li et al., 2022; Li et al., 2015) documented no adverse events, while no relevant safety data were provided in the remaining studies.

### Risk of bias assessment

**Figure 2:**
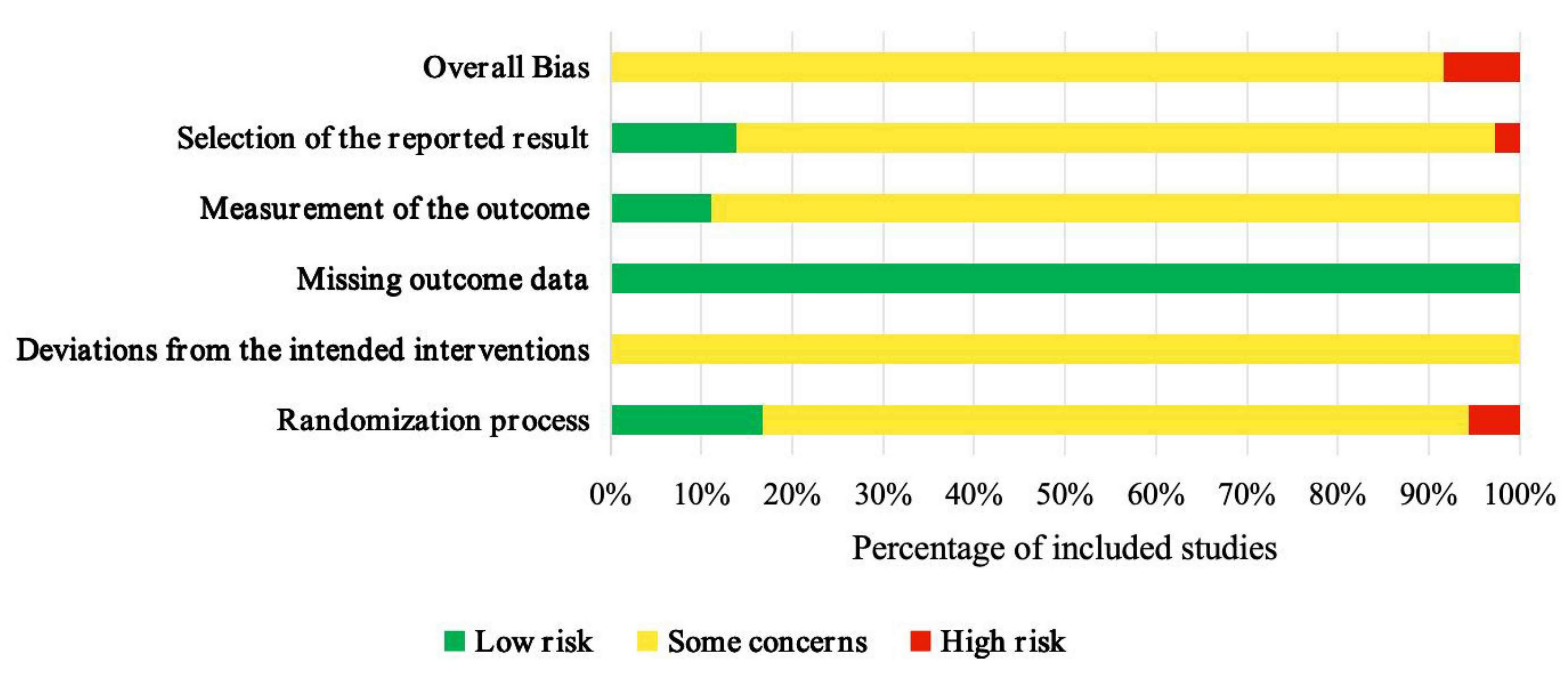
Risk-of-bias graph of included studies.

**Figure 3:**
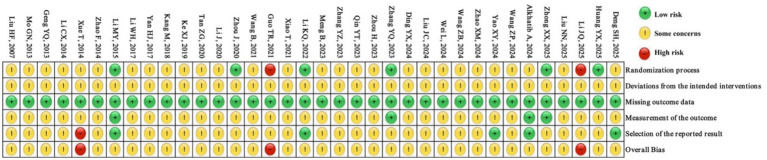
Risk-of-bias summary of included studies.

The methodological quality of the enrolled RCTs is visualized in Figure 2 (risk-of-bias graph) and Figure 3 (risk-of-bias summary). Regarding the randomization process domain, six studies (Huang, 2025; Zhong, 2025; Zhang and Jiang, 2023; Li et al., 2022; Zhou et al., 2020; Li et al., 2015) were rated “Low risk,” two studies (Li, 2025; Guo, 2021) “High risk,” and 28 studies “Some concerns” due to insufficient details on allocation concealment. Blinding of participants and providers was unfeasible given the nature of the Baduanjin exercise, resulting in “Some concerns” for all studies in the deviations from the intended interventions domain. All studies were rated “Low risk” for missing outcome data. In the outcome measurement domain, four studies (Zhong, 2025; Alkhatib et al., 2024; Zhang and Jiang, 2023; Li et al., 2015) were rated “Low risk” (due to assessor blinding), while the remaining studies were rated “Some concerns.” For the selection of the reported results domain, five studies (Deng, n.d.; Alkhatib et al., 2024; Yao et al., 2024; Li et al., 2022; Li et al., 2015) were rated “Low risk” (protocol pre-registration), one study (Xue et al., 2014) “High risk” (outcome inconsistency), and the rest “Some concerns”.

### Results of meta-analysis

#### SCL-90

**Figure 4:**
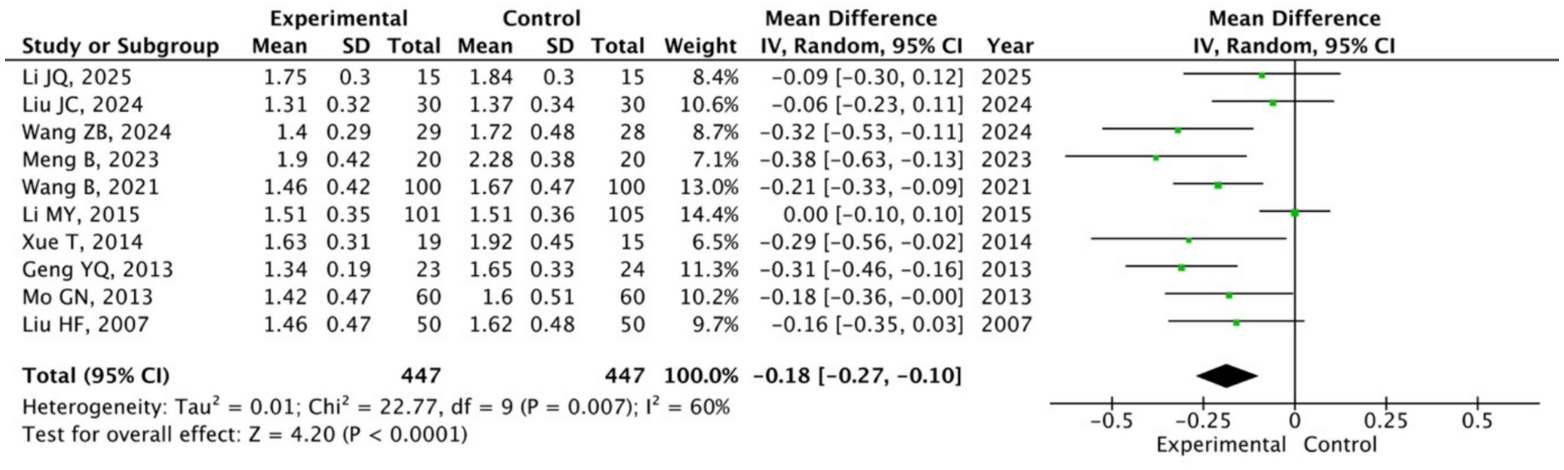
Pooled mean scores of SCL-90 in Baduanjin versus control groups.

A total of 16 studies (Li, 2025; Wang Z. P. et al., 2024; Zhao et al., 2024; Wang, 2025; Liu et al., 2024; Zhang and Jiang, 2023; Meng, 2023; Guo, 2021; Wang et al., 2021; Kang et al., 2018; Yan and Wei, 2017; Li et al., 2015; Xue et al., 2014; Geng, 2013; Mo, 2013; Liu, 2007) involving 1,406 participants reported SCL-90 outcomes. Among these, 10 studies (Li, 2025; Wang, 2025; Liu et al., 2024; Meng, 2023; Wang et al., 2021; Li et al., 2015; Xue et al., 2014; Geng, 2013; Mo, 2013; Liu, 2007) (*n* = 894) focused on overall mental health levels, while 15 studies (Li, 2025; Wang Z. P. et al., 2024; Zhao et al., 2024; Wang, 2025; Liu et al., 2024; Zhang and Jiang, 2023; Meng, 2023; Guo, 2021; Wang et al., 2021; Kang et al., 2018; Yan and Wei, 2017; Xue et al., 2014; Geng, 2013; Mo, 2013; Liu, 2007) (*n* = 1,200) reported dimension-specific scores. Random-effects model analysis showed that the Baduanjin group had a significantly lower overall mean SCL-90 score compared with the control group (MD = −0.18, 95% CI: −0.27 to −0.10, *p* < 0.0001; Figure 4). Except for interpersonal sensitivity and additional items in the unhealthy subgroup of university students, all other SCL-90 dimensions, including somatization, depression, anxiety, and interpersonal sensitivity, exhibited significant reductions in the Baduanjin group (all *p* < 0.05; Table 3). These results confirm that Baduanjin effectively reduces various psychological symptoms and improves the overall mental health status of university students.

**Table 3:** Subgroup analysis of Baduanjin’s effects by health status and scale subscales.

Subgroup	Health status	Number of studies	Total sample size	Heterogeneity		Effect size (95% CI)	*p*-value
				P	I^2^		
SCL-90	Healthy	7	783	0.07	49%	−0.21 (−0.30 to −0.13)	*p*<0.0001
	Unhealthy	3	111	0.25	29%	−0.25 (−0.36 to −0.13)	*p*<0.0001
SDS	Healthy	3	237	0.26	26%	−4.53 (−5.06 to −4.00)	*p*<0.0001
	Depression	3	185	0.01	77%	−4.01 (−7.03 to −0.99)	*p* = 0.0009
SAS	Healthy	2	200	0.02	83%	−4.89 (−9.02 to −0.76)	*p* = 0.02
	Unhealthy	2	112	0.89	0%	−2.88 (−5.30 to −0.45)	*p* = 0.02
PSQI	Healthy	3	328	0	96%	−0.90 (−1.06 to −0.73)	*p*<0.0001
	Sleep disturbance	5	222	0	82%	−3.35 (−3.72 to −2.98)	*p*<0.0001
	Depression	3	355	0.22	34%	−1.63 (−2.19 to −1.07)	*p*<0.0001
	Discomfort	2	104	0.001	91%	−3.53 (−4.42 to −2.64)	*p*<0.0001
SCL-90 SUBSCALES							
Somatization	Healthy	10	870	0.05	47%	−0.09 (−0.14 to −0.04)	*p* = 0.0004
	Unhealthy	3	170	0.18	42%	−0.26 (−0.38 to −0.14)	*p*<0.0001
Obsessive-compulsive symptoms	Healthy	10	870	0.48	0%	−0.18 (−0.24 to −0.12)	*p*<0.0001
	Unhealthy	3	170	0.68	0%	−0.35 (−0.54 to −0.17)	*p* = 0.0002
Interpersonal sensitivity	Healthy	10	870	0.07	43%	−0.14 (−0.20 to −0.09)	*p*<0.0001
	Unhealthy	3	170	0.006	80%	−0.35 (−0.76 to −0.05)	*p* = 0.09
Depression	Healthy	10	870	0.32	13%	−0.23 (−0.29 to −0.18)	*p*<0.0001
	Unhealthy	5	330	0.37	6%	−0.27 (−0.35 to −0.18)	*p*<0.0001
Anxiety	Healthy	10	870	0.59	0%	−0.17 (−0.22 to −0.12)	*p*<0.0001
	Unhealthy	4	296	0.01	72%	−0.28 (−0.46 to −0.10)	*p* = 0.002
Hostility	Healthy	10	870	0.08	42%	−0.12 (−0.18 to −0.07)	*p*<0.0001
	Unhealthy	4	204	0.65	0%	−0.21 (−0.36 to −0.07)	*p* = 0.005
Terror	Healthy	10	870	0.42	2%	−0.10 (−0.16 to −0.05)	*p* = 0.0002
	Unhealthy	4	204	0.07	57%	−0.26 (−0.48 to −0.03)	*p* = 0.03
Paranoid ideation	Healthy	10	870	0.001	67%	−0.22 (−0.32 to −0.11)	*p*<0.0001
	Unhealthy	3	170	0.69	0%	−0.28 (−0.45 to −0.11)	*p* = 0.002
Psychoticism	Healthy	10	870	0.07	43%	−0.15 (−0.20 to −0.10)	*p*<0.0001
	Unhealthy	4	204	0.29	0%	−0.22 (−0.36 to −0.08)	*p* = 0.002
Additional items	Healthy	8	737	0.12	39%	−0.14 (−0.20 to −0.08)	*p*<0.0001
	Unhealthy	3	170	0.01	78%	−0.33 (−0.67 to 0.02)	*p* = 0.07
POMS subscales							
Negative mood dimensions							
Tension-anxiety	Healthy	3	500	0.87	0%	−1.22 (−1.86 to −0.57)	*p* = 0.0002
Anger-hostility	Healthy	3	500	0.71	0%	−1.09 (−1.78 to −0.40)	*p* = 0.002
Depression-dejection	Healthy	3	500	0.97	0%	−1.22 (−1.98 to −0.46)	*p* = 0.002
Confusion-bewilderment	Healthy	3	500	0.42	0%	−1.24 (−1.82 to −0.66)	*p*<0.0001
Fatigue-inertia	Healthy	3	500	0.52	0%	−0.75 (−1.32 to −0.18)	*p* = 0.01
Positive mood dimensions							
Vigor-activity	Healthy	3	500	0.95	0%	0.44 (−0.45 to 1.33)	*p* = 0.33
Self-esteem	Healthy	3	500	0.82	0%	0.71 (0.09 to 1.32)	*p* = 0.03
DASS-21 Subscales							
Depression	Mixed	2	97	0.17	47%	−1.44 (−2.55 to −0.33)	*p* = 0.01
Anxiety	Mixed	2	97	0.76	0%	−2.83 (−3.68 to −1.97)	*p*<0.0001
Stress	Mixed	2	97	0.11	61%	−1.52 (−3.30 to 0.26)	*p* = 0.09
Follow-up data							
PSQI	Mixed	2	256	0.0001	94%	−1.41 (−3.81 to 0.99)	*p* = 0.25
PSS	Mixed	3	321	0.008	79%	−0.45 (−0.99 to 0.09)	*p* = 0.10

#### SDS and SAS

**Figure 5:**
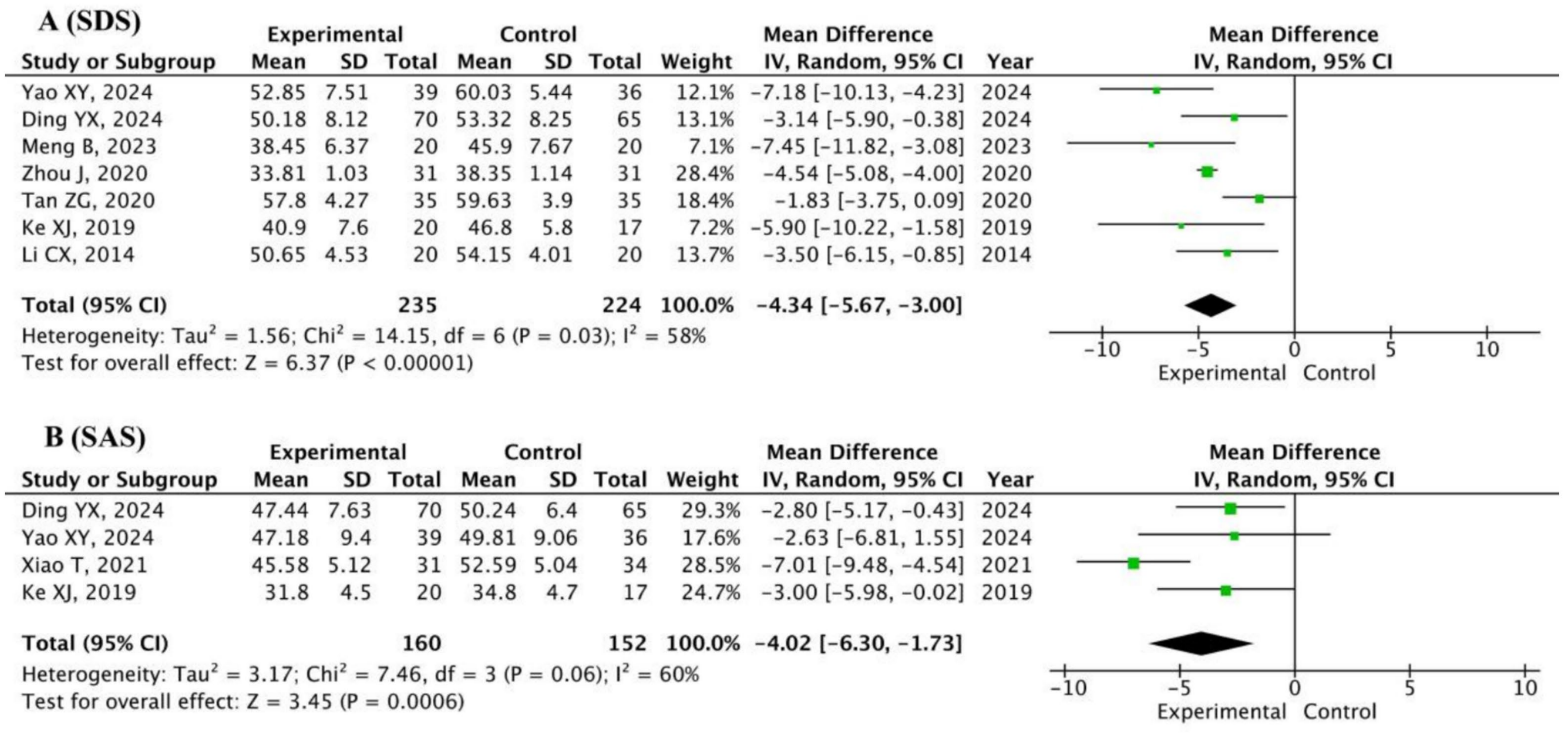
Pooled scores of SDS **(A)** and SAS **(B)** in Baduanjin versus control groups.

A total of seven studies (*n* = 459) employing the SDS (Yao et al., 2024; Ding and Tan, 2024; Meng, 2023; Zhou et al., 2020; Tan and Tan, 2020; Ke et al., 2019; Li et al., 2014), and four studies (*n* = 312) using the SAS (Yao et al., 2024; Ding and Tan, 2024; Xiao et al., 2021; Ke et al., 2019) were subjected to random-effects model analysis. Compared with the control group, the Baduanjin group exhibited significantly lower SDS scores (MD = −4.34, 95% CI: −5.67 to −3.00, *p* < 0.0001; Figure 5) and SAS scores (MD = −4.02, 95% CI: −6.30 to −1.73, *p* = 0.0006; Figure 5). These results confirm that Baduanjin effectively reduces depressive and anxious symptoms among university students.

#### POMS

**Figure 6:**
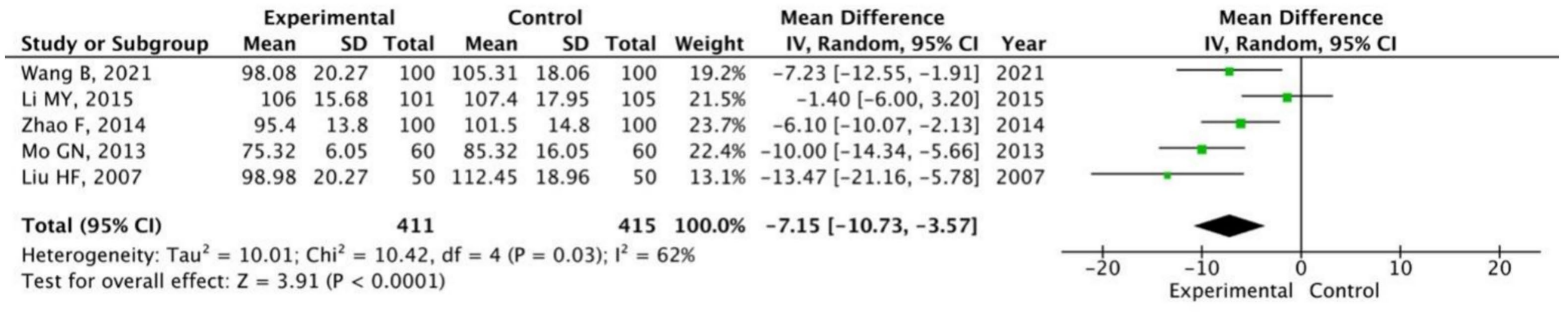
Pooled total scores of POMS in Baduanjin versus control groups.

Five studies (Wang et al., 2021; Li et al., 2015; Zhao and Li, 2014; Mo, 2013; Liu, 2007) (*n* = 826) assessed the POMS total score, while three studies (Wang et al., 2021; Zhao and Li, 2014; Liu, 2007) (*n* = 500) reported scores for its specific dimensions. All these studies were conducted among healthy university students. Random-effects model analysis indicated that the Baduanjin group had a significantly lower POMS total score than the control group (MD = −7.15, 95% CI: −10.73 to −3.57, *p* < 0.0001; Figure 6). Negative mood dimensions, such as tension-anxiety, depression-dejection, were significantly reduced (all *p* < 0.05; Table 3). No significant difference was observed in the positive dimension of vigor-activity; however, self-esteem showed a marginally significant improvement (*p* = 0.03). These findings indicate that Baduanjin effectively alleviates various negative emotions in university students, whereas the evidence for its effect on improving positive emotions is limited and inconsistent.

#### PSS/CPSS and DASS-21

**Figure 7:**
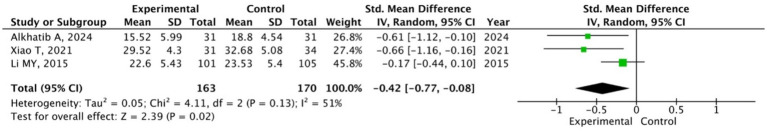
Pooled scores of PSS/CPSS in Baduanjin versus control groups.

Three studies (Alkhatib et al., 2024; Xiao et al., 2021; Li et al., 2015) (*n* = 333) using the PSS/CPSS were analyzed via a randon-effects model, which demonstrated that the Baduanjin group had a significantly lower stress score (SMD = −0.42, 95% CI: −0.77 to −0.08, *p* = 0.02; Figure 7). Two studies (Deng, n.d.; Zhang, 2023) (*n* = 97) using the DASS-21 revealed significant reductions in depressive and anxious symptoms in the Baduanjin group (both *p* < 0.05), but no significant effect on stress (Table 3). These findings suggest that Baduanjin effectively alleviates stress (assessed by PSS/CPSS), depression, and anxiety, with inconsistent results for stress measured by DASS-21.

#### PSQI and FS-14

**Figure 8:**
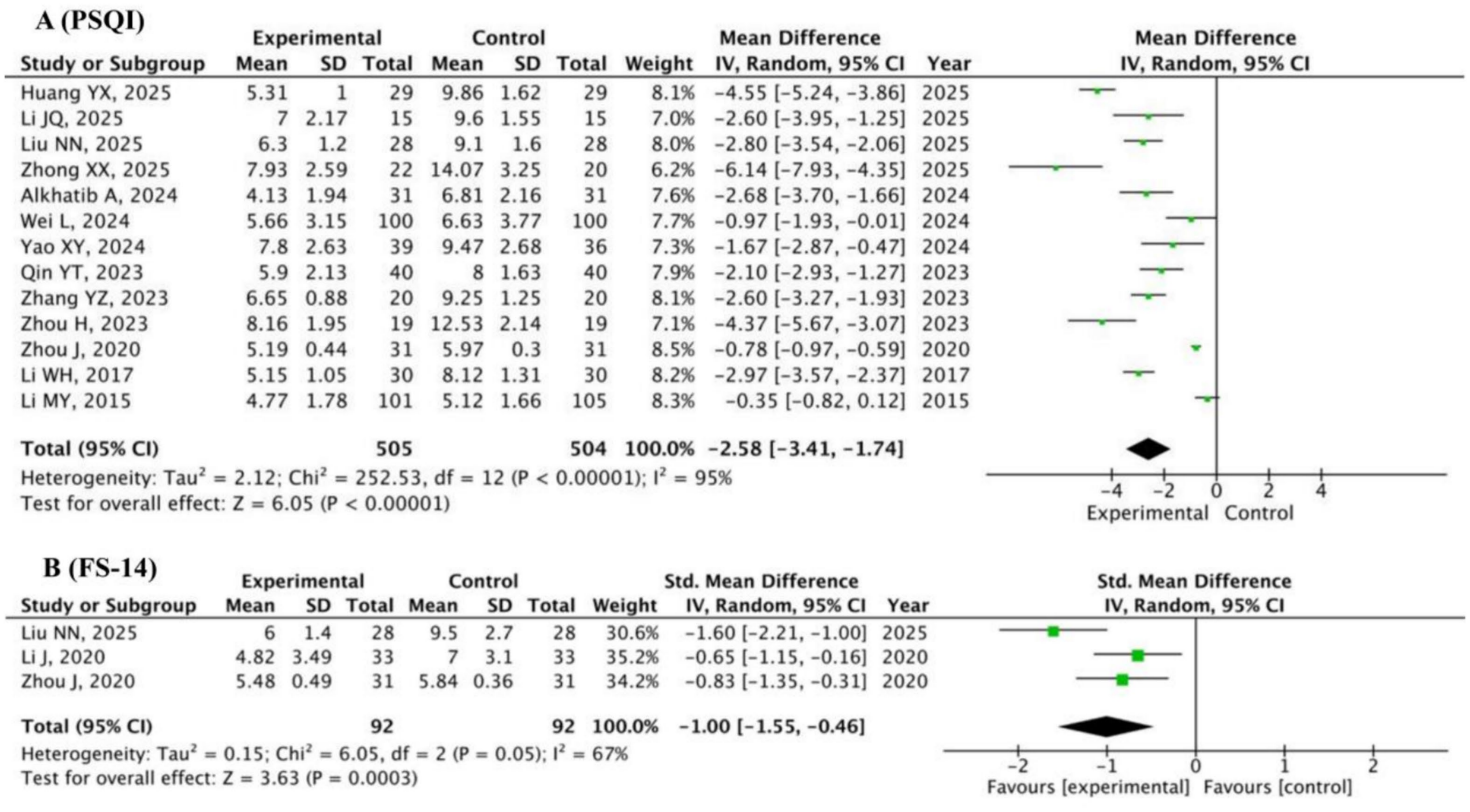
Pooled scores of PSQI **(A)** and FS-14 **(B)** in Baduanjin versus control groups.

**Figure 9:**
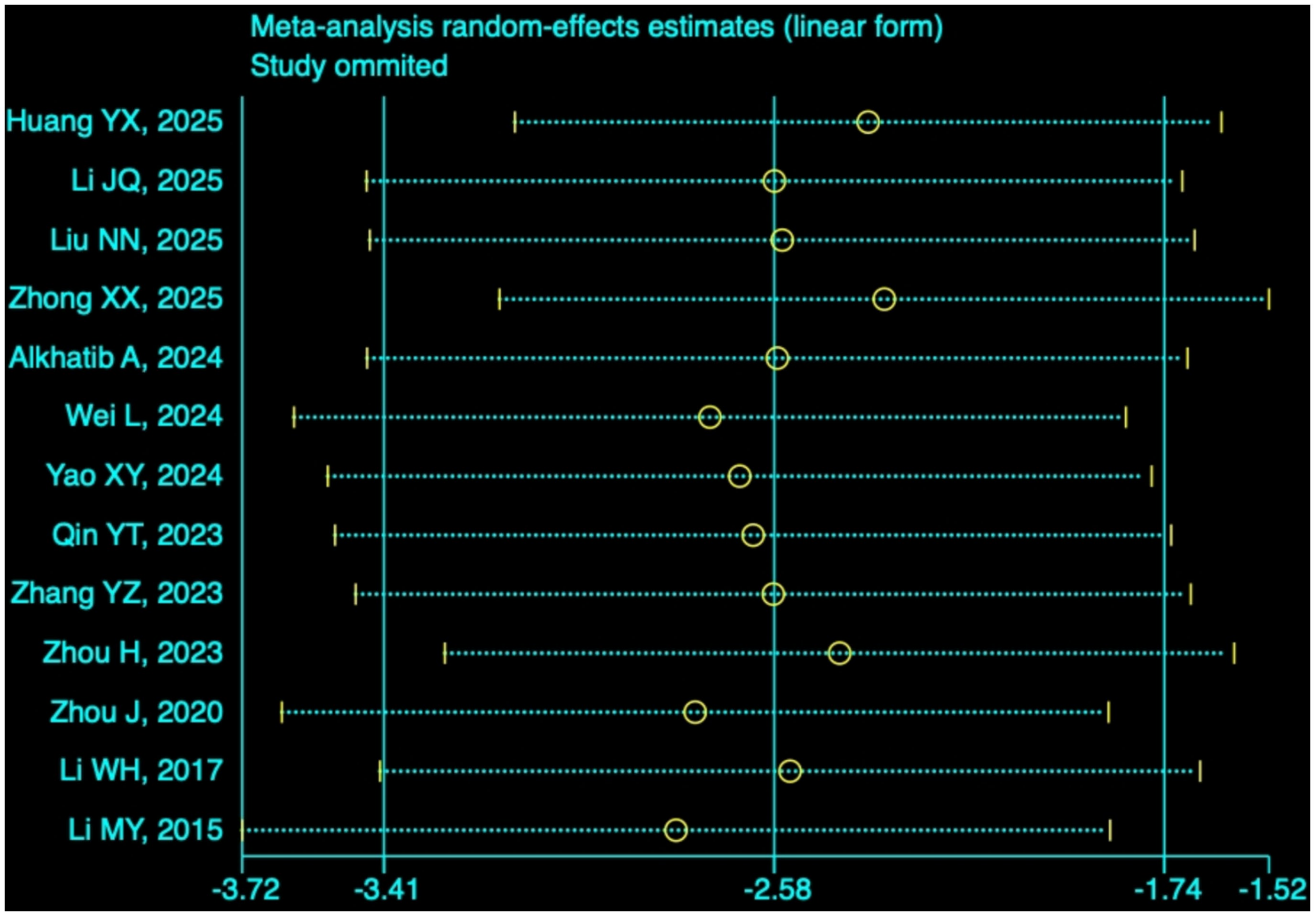
Sensitivity analysis of PSQI total scores.

A total of 13 studies (Huang, 2025; Li, 2025; Liu N. N. et al., 2025; Zhong, 2025; Alkhatib et al., 2024; Yao et al., 2024; Wei et al., 2024; Zou et al., 2023; Qin et al., 2023; Zhang, 2023; Zhou et al., 2020; Li, 2017; Li et al., 2015) (*n* = 1,009) employing the PSQI and three studies (Liu N. N. et al., 2025; Zhou et al., 2020; Li et al., 2020) (*n* = 184) using the FS-14 were analyzed. The random-effects model showed that the Baduanjin group had a significantly lower PSQI score (MD = −2.58, 95% CI: −3.41 to −1.74, *p* < 0.0001; Figure 8), and the random-effects model revealed a significantly lower FS-14 score (SMD = −1.00, 95% CI: −1.55 to −0.46, *p* = 0.0003; Figure 8) than the control group. These results indicate that Baduanjin effectively enhances sleep quality and alleviates fatigue among university students. Sensitivity analysis for PSQI (Figure 9) showed that the pooled effect size remained largely unchanged after the stepwise exclusion of each individual study, with no outlier study identified. This further verifies the robustness of the conclusion that Baduanjin improves university students’ sleep quality.

#### Follow-up analysis (PSQI and PSS)

Follow-up data for the PSQI [two studies (Alkhatib et al., 2024; Li et al., 2015), *n* = 256] and PSS [three studies (Alkhatib et al., 2024; Xiao et al., 2021; Li et al., 2015), *n* = 321] were analyzed using random-effects models. No significant differences were observed between the Baduanjin and control groups in PSQI (MD = −1.41, 95% CI: −3.81 to 0.99, *p* = 0.25) or PSS scores (SMD = −0.45, 95% CI: −0.99 to 0.09, *p* = 0.10) at follow-up (Table 3). These findings suggest that the beneficial effects of Baduanjin on sleep quality and stress may not be sustained over time.

#### Subgroup analysis

Subgroup analysis was performed to explore the associations between intervention parameters and outcomes, stratified by intervention duration, weekly frequency, and session length (Table 4). In the present analysis, weekly frequency appeared to be a potential moderator of SCL-90-lowering efficacy (*p* = 0.003, I^2^ = 82.9%), with three sessions per week yielding the greatest benefit. Total intervention duration also emerged as a potential moderator for depressive symptom reduction (SDS), favoring interventions of 12 weeks or less (*p* = 0.004, I^2^ = 87.6%). No significant subgroup differences were observed for any other stratifications, including SCL-90 total and session duration, SDS weekly frequency, and session length (all *>* 0.05). It should be noted that some subgroup findings were based on a limited number of studies, potentially affecting their robustness. For sleep quality (PSQI) and SCL-90 depression/anxiety dimensions, no statistically significant subgroup differences were detected across the parameters tested. This suggests that the beneficial effects on these outcomes may be relatively stable across the range of parameters examined in the included studies.

**Table 4:** Subgroup analysis of Baduanjin’s effects on SCL-90, SDS, and PSQI stratified by intervention parameters.

Moderating variable	Stratified subgroup	Number of studies	Total sample size	Within-subgroup heterogeneity		Effect size (95% CI)	*p* value	Test for subgroup differences	
				*p*	I^2^			*p*	I^2^
SCL-90									
Intervention duration	<12 weeks	2	81	0.9	0%	−0.31 (−0.44, −0.17)	*p*<0.0001	0.2	38.60%
	= 12 weeks	5	433	0.008	71%	−0.17 (−0.32, −0.02)	*p* = 0.02		
	>12 weeks	3	380	0.36	3%	−0.16 (−0.25, −0.07)	*p* = 0.0003		
Weekly frequency	<3 sessions/week	2	64	0.25	24%	−0.17 (−0.34, −0.00)	*p* = 0.05	0.003	82.90%
	= 3 sessions/week	6	524	0.18	34%	−0.23 (−0.29, −0.16)	*p*<0.0001		
	>3 sessions/week	2	306	0.14	55%	−0.03 (−0.12, 0.05)	*p* = 0.44		
Session length	≤ 60 min	5	633	0.005	73%	−0.15 (−0.27, −0.03)	*p* = 0.02	0.28	15%
	>60 min	5	261	0.34	11%	−0.23 (−0.34, −0.13)	*p*<0.0001		
SCL-90-depression									
Intervention duration	<12 weeks	2	81	0.96	0%	−0.28 (−0.44, −0.11)	*p* = 0.001	0.12	53.40%
	= 12 weeks	8	586	0.47	0%	−0.27 (−0.33, −0.22)	*p*<0.0001		
	>12 weeks	5	533	0.39	2%	−0.16 (−0.25, −0.08)	*p* = 0.0002		
Weekly frequency	<3 sessions/week	2	64	0.34	0%	−0.14 (−0.34, 0.06)	*p* = 0.16	0.56	0%
	= 3 sessions/week	10	876	0.29	17%	−0.25 (−0.30, −0.19)	*p*<0.0001		
	>3 sessions/week	3	260	0.34	6%	−0.26 (−0.35, −0.17)	*p*<0.0001		
Session length	<60 min	4	267	0.22	32%	−0.25 (−0.32, −0.17)	*p*<0.0001	0.5	0%
	= 60 min	5	472	0.39	4%	−0.27 (−0.35, −0.19)	*p*<0.0001		
	>60 min	6	461	0.41	1%	−0.20 (−0.29, −0.11)	*p*<0.0001		
SCL-90-anxiety									
Intervention duration	= 12 weeks	8	586	0.28	19%	−0.20 (−0.26, −0.14)	*p*<0.0001	0.87	0%
	>12 weeks	5	533	0.05	57%	−0.19 (−0.32, −0.06)	*p* = 0.005		
Weekly frequency	= 3 sessions/week	10	876	0.08	42%	−0.23 (−0.29, −0.18)	*p*<0.0001	0.12	58.60%
	>3 sessions/week	3	260	0.9	0%	−0.15 (−0.24, −0.07)	*p* = 0.0003		
Session length	<60 min	4	267	0.04	63%	−0.18 (−0.31, −0.05)	*p* = 0.006	0.64	0%
	= 60 min	5	472	0.23	29%	−0.24 (−0.33, −0.15)	*p*<0.0001		
	>60 min	5	427	0.24	28%	−0.18 (−0.29, −0.07)	*p* = 0.001		
SDS									
Intervention duration	≤12 weeks	4	214	0.18	38%	−4.69 (−5.21, −4.16)	*p*<0.0001	0.004	87.60%
	>12 weeks	3	245	0.55	0%	−2.58 (−3.93, −1.23)	*p* = 0.0002		
Weekly frequency	= 3 sessions/week	2	175	0.1	63%	−4.95 (−9.12, −0.78)	*p* = 0.02	0.63	0%
	= 5 sessions/week	3	169	0.02	74%	−3.80 (−5.95, −1.65)	*p* = 0.0005		
Session length	<60 min	4	342	0.01	74%	−4.05 (−5.86, −2.24)	*p*<0.0001	0.51	0%
	≥ 60 min	3	117	0.27	23%	−5.06 (−7.43, −2.68)	*p*<0.0001		
PSQI									
Intervention duration	4–8 weeks	4	237	0.0001	96%	−2.00 (−3.33, −0.67)	*p* = 0.003	0.12	52.90%
	10 weeks	3	152	0.002	84%	−3.88 (−5.12, −2.64)	*p*<0.0001		
	12 weeks	5	420	0.0001	93%	−2.64 (−4.23, −1.04)	*p* = 0.001		
Weekly frequency	<5 sessions/week	5	390	0.0001	88%	−3.12 (−4.66, −1.58)	*p*<0.0001	0.62	0%
	= 5 sessions/week	6	482	0.0001	97%	−2.32 (−3.54, −1.10)	*p* = 0.0002		
	>5 sessions/week	2	137	0.21	36%	−2.23 (−3.21, −1.24)	*p*<0.0001		
Session length	<60 min	6	357	0.0001	95%	−2.71 (−4.00, −1.42)	*p*<0.0001	0.57	0%
	= 60 min	5	422	0.0001	97%	−2.76 (−4.48, −1.05)	*p* = 0.002		
	>60 min	2	230	0.05	73%	−1.71 (−3.30, −0.12)	*p* = 0.03		

Subgroup analyses stratified by baseline health status and control conditions were conducted to verify the robustness of the findings (Table 3; Table 5). For baseline health status, Baduanjin significantly improved SCL-90, SDS, SAS, and PSQI in both healthy and unhealthy students, including those with depression, sleep disturbance, and physical discomfort (all *p* < 0.05). For control conditions, compared with the usual study/lifestyle, Baduanjin significantly improved all SCL-90 subscales (all *p* < 0.001). Compared with extracurricular exercise, Baduanjin still significantly reduced depression, anxiety, and psychoticism (all *p* < 0.05), with no significant between-group differences in other subscales.

**Table 5:** Subgroup analysis of Baduanjin’s effects on SCL-90 subscales stratified by control conditions.

Subgroup	Control conditions	Number of studies	Total sample size	Heterogeneity		Effect size (95% CI)	*p*-value
				*p*	I^2^		
Somatization	Usual study/lifestyle	11	920	0.04	48%	−0.15 (−0.21 to −0.10)	*p*<0.0001
	Extracurricular exercise	2	120	0.76	0%	−0.00 (−0.10 to 0.09)	*p* = 0.97
Obsessive-compulsive symptoms	Usual study/lifestyle	11	920	0.63	0%	−0.22 (−0.28 to −0.16)	*p*<0.0001
	Extracurricular exercise	2	120	0.71	0%	−0.05 (−0.20 to 0.10)	*p* = 0.51
Interpersonal sensitivity	Usual study/lifestyle	11	920	0.002	63%	−0.20 (−0.31 to −0.10)	*p* = 0.0002
	Extracurricular exercise	2	120	1	0%	−0.10 (−0.22 to 0.02)	*p* = 0.10
Depression	Usual study/lifestyle	12	954	0.42	2%	−0.25 (−0.30 to −0.20)	*p*<0.0001
	Extracurricular exercise	3	246	0.18	43%	−0.22 (−0.31 to −0.14)	*p*<0.0001
Anxiety	Usual study/lifestyle	11	920	0.11	36%	−0.20 (−0.25 to −0.15)	*p*<0.0001
	Extracurricular exercise	3	246	0.05	67%	−0.16 (−0.31 to −0.01)	*p* = 0.03
Hostility	Usual study/lifestyle	12	954	0.29	16%	−0.16 (−0.21 to −0.10)	*p*<0.0001
	Extracurricular exercise	2	120	0.15	53%	−0.02 (−0.24 to 0.19)	*p* = 0.84
Terror	Usual study/lifestyle	12	954	0.12	33%	−0.14 (−0.19 to −0.08)	*p*<0.0001
	Extracurricular exercise	2	120	0.74	0%	−0.03 (−0.15 to 0.08)	*p* = 0.59
Paranoid ideation	Usual study/lifestyle	11	920	0.005	61%	−0.25 (−0.36 to −0.15)	*p*<0.0001
	Extracurricular exercise	2	120	0.45	0%	−0.10 (−0.23 to 0.03)	*p* = 0.14
Psychoticism	Usual study/lifestyle	12	954	0.05	43%	−0.16 (−0.22 to −0.11)	*p*<0.0001
	Extracurricular exercise	2	120	0.35	0%	−0.14 (−0.26 to −0.03)	*p* = 0.02
Additional items	Usual study/lifestyle	9	787	0.009	60%	−0.21 (−0.32 to −0.11)	*p*<0.0001
	Extracurricular exercise	2	120	0.51	0%	−0.09 (−0.21 to 0.03)	*p* = 0.13

### Heterogeneity assessment

The meta-analysis revealed beneficial effects of Baduanjin exercise on the mental health of university students; however, significant heterogeneity was observed in the pooled effect estimates for several primary outcomes. To investigate potential sources of heterogeneity, analyses were conducted from both clinical and methodological perspectives. Clinical heterogeneity was primarily reflected in: (1) diverse baseline health statuses of participants, including healthy individuals and those with conditions such as depression or sleep disorders; (2) considerable variation in intervention protocols regarding duration, frequency, and session length; and (3) differing control conditions, ranging from passive usual care to active exercise interventions. Although subgroup analyses were performed, high residual heterogeneity persisted within some subgroups, suggesting the potential influence of unmeasured confounding factors or complex interactions among intervention parameters. Methodological heterogeneity mainly involved: (1) inadequate reporting or implementation of allocation concealment and blinding of outcome assessors in most studies, and (2) variations in the timing of outcome assessments and measurement tools. These factors may introduce bias and increase variability in effect estimates.

Notably, despite the observed heterogeneity, the positive effects of Baduanjin on most mental health outcomes remained statistically significant under the random-effects model, supporting the robustness of the primary findings. Nevertheless, the presence of heterogeneity indicates that intervention effects may be moderated by participant characteristics and implementation details, warranting cautious interpretation of the effect size magnitudes. In particular, results from certain subgroup analyses and long-term follow-up assessments, which were based on a limited number of studies with substantial heterogeneity, require further validation through more high-quality research.

### Publication bias

**Figure 10:**
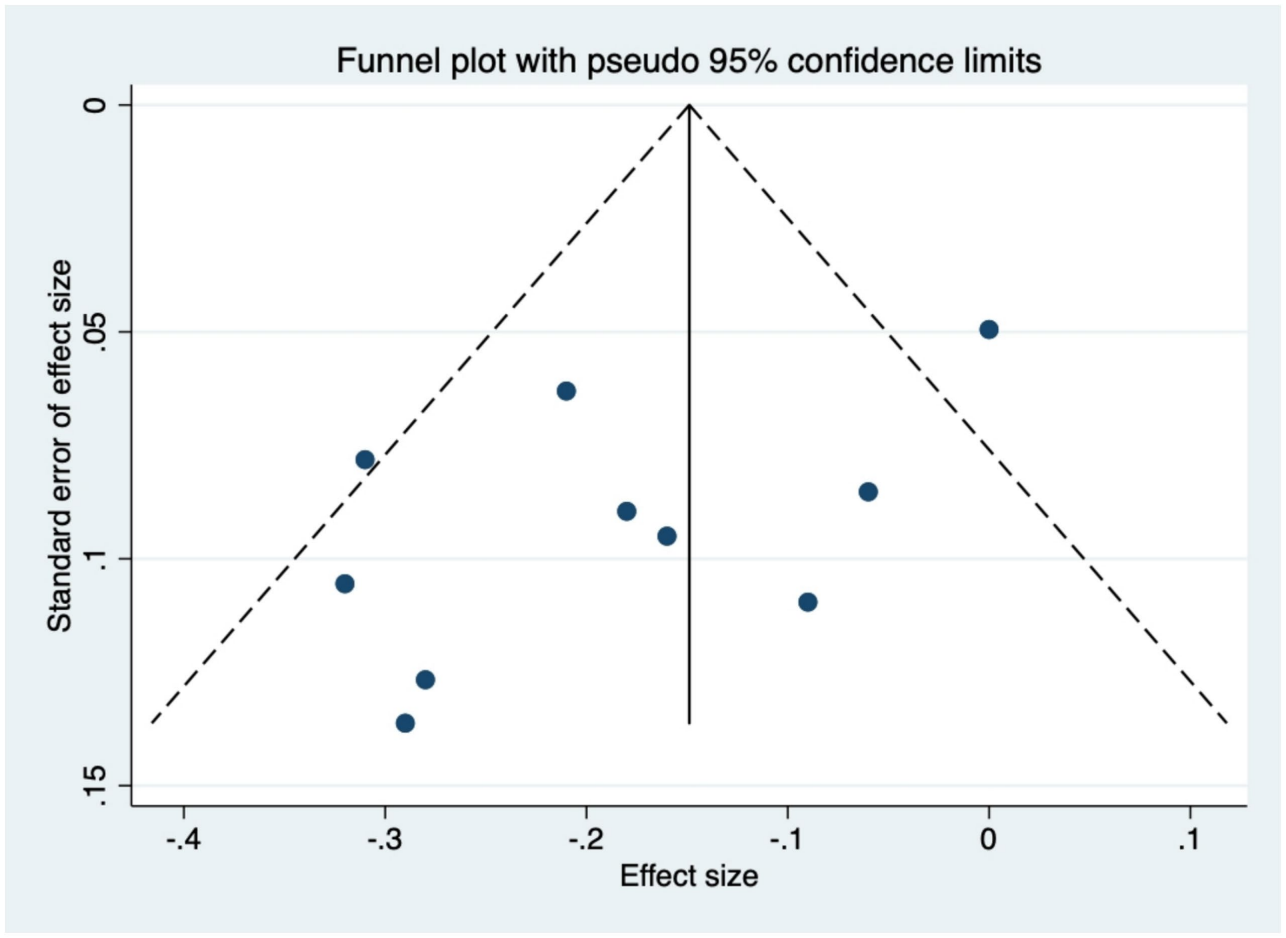
Funnel plot for publication bias of SCL-90 total scores.

Publication bias was evaluated via funnel plot analysis of 10 studies (Li, 2025; Wang, 2025; Liu et al., 2024; Meng, 2023; Wang et al., 2021; Li et al., 2015; Xue et al., 2014; Geng, 2013; Mo, 2013; Liu, 2007) reporting SCL-90 outcomes. The results demonstrated a symmetrical distribution of the funnel plot (Figure 10); Egger’s test revealed no statistically significant publication bias in this study (*p* = 0.07). These results showed that the conclusions of this systematic review and meta-analysis were minimally affected by publication bias, supporting their high robustness and reliability.

### Safety analysis

Regarding safety, only three of the 36 included studies reported adverse reactions related to Baduanjin exercise, and only one study described the monitoring method and follow-up process of adverse events in detail. The scarcity of safety data considerably limits a systematic and objective assessment of the potential risks of Baduanjin intervention. In clinical practice, low incidence and mild adverse events, such as muscle soreness or transient discomfort resulting from improper movement patterns, are easily overlooked by researchers and may be missed, underreported, or even not actively collected. Consequently, the current evidence supporting the safety of Baduanjin lacks high-quality and systematic empirical support, and its potential risks may be underestimated.

## Discussion

### Key findings and interpretation

This systematic review and meta-analysis integrated the evidence from 36 RCTs, including 3,233 university students, and comprehensively evaluated the effects of Baduanjin exercise on the mental health, sleep quality, and fatigue status of university students. The findings demonstrated that Baduanjin can significantly enhance the overall mental health of university students, effectively relieve negative emotions such as depression, anxiety, and stress, and notably improve sleep quality and reduce fatigue symptoms. Subgroup analyses stratified by baseline health status indicated that Baduanjin may yield benefits among both healthy students and those with psychological or somatic symptoms. Further subgroup analyses by control conditions demonstrated that Baduanjin provided consistent mental health benefits relative to both usual care and extracurricular exercise, suggesting its unique advantages in emotional regulation beyond general physical activity. In addition, a more favorable intervention effect on specific outcome indicators occurs when practiced three times a week, with a total intervention duration not exceeding 12 weeks. Although some subgroups were limited by small sample sizes, these preliminary findings may provide supportive evidence for the application of Baduanjin in promoting mental health among university students.

Baduanjin exhibits extensive intervention effects on mental health. The findings of the present study align with those of prior investigations among patients with chronic diseases (Kong et al., 2022) and the relevant evidence is extended to the university student population for the first time. The students are exposed to considerable academic pressure and developmental adaptation challenges, placing them in a high-risk group of psychological distress (Paiva et al., 2025). In terms of relieving depression and anxiety symptoms, the intervention effect of Baduanjin is comparable to that of low-intensity aerobic exercise (Zhang et al., 2024), and superior to mindfulness meditation among young individuals (Lothes et al., 2025). This advantage may stem from Baduanjin’s organic integration of somatic movement, respiratory regulation, and mindfulness awareness, which simultaneously acts on both physiological and psychological stress pathways, whereas meditation primarily relies on cognitive regulation as its core mechanism (García-Pérez et al., 2025). Although CBT demonstrates larger effect sizes for depressive disorder (Chan et al., 2025), its accessibility is significantly constrained. By contrast, Baduanjin produces comparable intervention efficacy for mild-to-moderate psychological symptoms without additional cost or professional training, making it a first-line intervention for university students. Notably, the efficacy of Baduanjin in improving positive emotions is limited, a pattern similarly observed in yoga intervention studies (Xue et al., 2025), suggesting that mind–body exercise may predominantly focus on the reduction of negative emotions rather than the enhancement of positive emotional experiences.

Baduanjin effectively improved sleep quality, which is consistent with findings from previous meta-analyses in elderly populations with insomnia (Liang et al., 2024). This effect may be mediated through bidirectional regulatory pathways: the gentle movements and abdominal breathing of Baduanjin can attenuate sympathetic nervous system hyperactivity (Liu J. H. et al., 2023), thereby alleviating pre-sleep anxiety and stabilizing circadian rhythm. In turn, enhanced sleep quality can reduce daytime fatigue and enhance exercise adherence (Feng et al., 2025), which aligns with research demonstrating Baduanjin’s efficacy in alleviating fatigue symptoms in chronic disease patients (Liu H. Y. et al., 2023). However, follow-up analysis revealed that the gains in sleep quality and stress-related outcomes gradually diminished following intervention cessation, showing a notable contrast with the sustained sleep quality improvements observed with CBT for up to 12 months post-intervention (Wang H. J. et al., 2024). This finding highlights a key limitation of exercise interventions: Their effects are highly dependent on sustained physical activity, which is often difficult to maintain in a university student population because of heavy academic pressure (Pan et al., 2022). Evidence-based behavior change strategies, such as CBT-based habit formation approaches (Li and Tang, 2024) or structured peer support mechanisms (Lv et al., 2025), can be integrated to improve long-term exercise adherence and consolidate intervention effects.

Subgroup analysis further provided preliminary evidence regarding the potential effects of intervention parameters. Overall mental health (SCL-90) improved most prominently at the frequency of three sessions per week, with no additional benefit observed at higher frequencies. This finding is consistent with research on aerobic exercise, suggesting that moderate training frequency helps prevent physical fatigue and improves adherence (Wang and Tang, 2024). Nevertheless, the above interpretation should be interpreted with caution due to the limited number of studies included in the higher-frequency subgroup. Intervention durations of up to 12 weeks were superior to longer interventions with respect to improvement in depressive symptoms. Should this result be corroborated by further research, it would challenge the conventional wisdom that longer interventions are better (Zhang et al., 2024). This phenomenon may be attributed to diminished practice quality during the long-term intervention, such as reduced motivation or disruptions from daily life events, or may reflect the rapid stress relief effect of Baduanjin itself, which aligns well with the short-term nature of academic stress among university students. Notably, the sleep-improving effects of Baduanjin remained stable across variations in intervention frequency or duration. Such robustness is particularly critical for university students with irregular work and fragmented time availability, as it allows them to flexibly arrange practice within a limited time without compromising the intervention efficacy. Meanwhile, subgroup analyses stratified by health status and control type showed that Baduanjin significantly improved mental health, depression, anxiety, and sleep outcomes in both healthy university students and those with psychological or somatic symptoms. Compared with usual care, Baduanjin comprehensively enhanced all dimensions of mental health. Compared with other extracurricular physical exercises, Baduanjin still exhibited unique advantages in improving depressive, anxiety, and psychotic symptoms, indicating that its emotion-regulating effects are not merely derived from physical activity but involve specific benefits of mind–body regulation.

In summary, the subgroup analyses of this study suggest promising optimized protocols. However, given the small sample size in some subgroups, the presence of certain heterogeneity, and the exploratory nature of these analyses, the above optimal parameters should be interpreted prudently. They represent promising directions for future research rather than definitive exercise prescriptions.

### Methodological strengths and limitations

The overall methodological quality of the RCTs included in this study was moderate, and their strengths and limitations need to be interpreted cautiously. Key strengths included the large sample size (3,233 participants) and the use of multiple psychometrically validated assessment tools, which enhanced the robustness of the results. Neither sensitivity analysis nor Egger’s test suggests significant publication bias, which further enhances the credibility of this meta-analysis. However, several methodological limitations remained. First, specific ways in which allocation concealment was implemented were underreported in 77.8% of the studies, a more prominent problem than observed in the previous meta-analysis of aerobic exercise or resistance training (59% of high-risk studies did not specify this) (Banyard et al., 2025). Second, only 11.1% of studies used blinded outcome assessors, which may increase the risk of detection bias for subjective outcome measures. Third, all eligible studies were conducted in mainland China, introducing geographical limitations that may affect the external generalization of the results. Fourth, most studies did not systematically report adverse events or provide long-term follow-up data, which limits the comprehensive evaluation of the safety and long-term efficacy of interventions. Fifth, some subgroup analyses included only two or three studies, which resulted in a weak evidence base and limited the reliability of the corresponding conclusions. Sixth, there is significant clinical and methodological heterogeneity among the studies, which limits the determination of optimal intervention parameters and suggests that the intervention effects may vary depending on specific implementation conditions and population characteristics. Consequently, although the main conclusions remain robust, more standardized and high-quality research is needed in the future to enhance the precision of the evidence.

### Practical implications for university health promotion

Given its gentle movements, low learning threshold, and low cost, Baduanjin may serve as a potentially valuable complementary intervention that could be considered for inclusion in campus health programs, pending further high-quality evidence. Based on the findings of this study, we recommend integrating the Baduanjin system into the university’s physical education curriculum or conducting it as a regular extracurricular health promotion activity. The implementation of the optimized group-based intervention program- lasting 40–60 min per session, conducted three times a week for 8–12 weeks- has significantly improved students’ participation compliance and adherence rates. Furthermore, combining sleep hygiene education and stress management skills training with the core strategies of CBT is expected to enhance and prolong the long-term health benefits of Baduanjin intervention (Huang D. Q. et al., 2024). In addition, the adoption of online teaching models, such as those effectively validated during the COVID-19 pandemic, can effectively improve the accessibility of the intervention for distance learners and students with time constraints (Lee et al., 2025), thereby further expanding the coverage and application depth of Baduanjin-based health intervention.

### Future research directions

Future research should prioritize addressing the limitations of the present study to further consolidate the evidence base. Multi-center studies encompassing different ethnic populations are recommended to reduce regional bias and improve the external validity and generalizability of the results. From a methodological perspective, more rigorous designs should be implemented, including standardized reporting of randomization and allocation concealment, blinding of outcome assessors, and prospective registration of study protocols on public platforms, to systematically reduce bias risk and improve overall research quality. Long-term follow-up studies with a minimum duration of 12 months are urgently required to fully evaluate the sustainability of the intervention effect. In addition, head-to-head randomized controlled trials with mainstream interventions such as CBT, aerobic exercise, and yoga will help clarify its comparative advantages and unique positioning of Baduanjin in the psychological intervention system of university students. Targeted research on specific high-risk groups, such as students with moderate to severe mental disorders, and exploration of combined intervention strategies like Baduanjin combined with CBT (Jing et al., 2018), will further expand the clinical applicability and practical value of this traditional mind–body exercise. Mechanism studies incorporating neuroimaging and neuroendocrine indicators (e.g., cortisol, brain-derived neurotrophic factor, etc.) are expected to reveal the physiological and psychological pathways of Baduanjin in regulating mental health, providing more convincing theoretical support for its scientific promotion.

## Conclusion

In conclusion, Baduanjin, as a simple yet evidence-based mind–body intervention, may effectively improve the mental health, sleep quality, and subjective fatigue of university students. Existing evidence indicates that interventions performed three times per week for up to 12 weeks may yield more favorable effects on certain outcomes. Given its strong universality, flexible implementation, and low cost, Baduanjin holds promise as a feasible complementary intervention strategy within colleges and universities’ mental health service systems, but their effects require confirmation in larger, high-quality studies. Future studies should adopt more rigorous designs to validate efficacy, sustainability, and safety, thereby maximizing its potential in alleviating the global mental health crisis among university students.

## Data Availability

The original contributions presented in the study are included in the article/Supplementary material, further inquiries can be directed to the corresponding authors.
