# Supplementary material for: Effectiveness of Baduanjin exercise for improving the mental health of university students: a systematic review and meta-analysis of randomized controlled trials
Source: Front Psychol. 2026 May 15;17:1825503. doi: 10.3389/fpsyg.2026.1825503 (PMC13218987; doi:10.3389/fpsyg.2026.1825503)
Supplement: Supplementary file 1 [file Supplementary_File_1.docx]

**Search Strategies**

**Web of Science:**

TS = (Baduanjin OR Ba Duan Jin OR Eight-Section Brocade OR Eight Section Brocades OR Eight Segments of Brocade) AND TS = (Students OR Pupil OR Schools OR College Student OR University Student OR Higher Education Institution OR Undergraduate OR Postgraduate) AND TS = (Depression OR Depressive Disorder OR Anxiety OR Anxiety Disorders OR Psychology OR Mood Disorders OR Emotions OR Mental Health OR Psychological Distress OR Stress OR Worry OR sleep OR sleep quality OR Sleep wake disorder OR dyssomnia OR insomnia OR rest OR break OR sleep debt OR fatigue OR Fatigue Syndrome, Chronic OR lethargy OR Chronic Fatigue Syndrome OR tiredness OR exhaustion OR weariness OR low energy)

**CNKI：**

(SU=八段锦+健身气功八段锦) and (SU=学生+大学生+高校+学校) and (SU=焦虑+抑郁+心理+情绪+心理健康+压力+睡眠+睡眠障碍+不寐+失眠+失眠症+疲劳+慢性疲劳综合征+CFS+疲倦+疲乏)
